# No evidence of secondary transmission of COVID-19 from children attending school in Ireland, 2020

**DOI:** 10.2807/1560-7917.ES.2020.25.21.2000903

**Published:** 2020-05-28

**Authors:** Laura Heavey, Geraldine Casey, Ciara Kelly, David Kelly, Geraldine McDarby

**Affiliations:** 1 Public Health Medicine, Health Service Executive, Dublin, Ireland; 2 These authors contributed equally to this article and share first authorship

**Keywords:** COVID-19, SARS-Cov2, school, paediatric, transmission

## Abstract

As many countries begin to lift some of the restrictions to contain COVID-19 spread, lack of evidence of transmission in the school setting remains. We examined Irish notifications of SARS-CoV2 in the school setting before school closures on 12 March 2020 and identified no paediatric transmission. This adds to current evidence that children do not appear to be drivers of transmission, and we argue that reopening schools should be considered safe accompanied by certain measures.

Coronavirus disease (COVID-19), which is caused by severe acute respiratory syndrome coronavirus 2 (SARS-CoV-2), was declared a pandemic on 11 March 2020 [1]. Many countries followed the precautionary principle and, to limit the spread of the virus, imposed restrictions on citizens, such as promoting physical distancing, limiting the movement of people, closing educational institutions and/or workplaces. Now countries, while continuing to control the spread of the virus, must plan how to lift some of these restrictions to allow people to resume activities of daily life.

Children are thought to be vectors for transmission of many respiratory diseases including influenza [2]. It was assumed that this would be true for COVID-19 also. To date however, evidence of widespread paediatric transmission has failed to emerge [3]. School closures create childcare issues for parents. This has an impact on the workforce, including the healthcare workforce [4]. There are also concerns about the impact of school closures on children’s mental and physical health [5].

We aimed to examine the evidence of paediatric transmission in the Republic of Ireland in the school setting.

## Irish school closures

The first Irish case of COVID-19 was notified in a school-going child who had recently returned from Northern Italy at the beginning of March 2020. As the numbers of cases detected in the community in Ireland began to increase, the National Public Health Emergency Team advised the closure of all schools from 12 March 2020 6 p.m., in an effort to contain the spread of COVID-19^.^[6].

## Finding coronavirus disease school-related cases and their contacts

To find evidence in the Republic of Ireland on COVID-19 transmissions related to schools before their closure, all SARS-CoV-2 notifications to Public Health Departments were screened to identify children, under the age of 18 years, and adults who had attended the school setting.

Cases were identified within the Computerised Infectious Disease Reporting (CIDR) system (Ireland’s national infectious disease surveillance system). On CIDR, attendance at work or school was routinely recorded for COVID-19 surveillance. Contact-tracing records and records from active surveillance were reviewed to identify cases of secondary transmission.

### Case descriptions

Three paediatric cases and three adult cases of COVID-19 with a history of school attendance were identified. The available epidemiological data for all of these cases indicated that they had not been infected with SARS-CoV-2 in the school setting. One case was travel related, while three cases were part of a single household outbreak, also linked to travel. One case was a close contact of a confirmed case in a recreational context, which was outside a school environment. One case was a contact of another case, and transmission occurred in a work environment.

One paediatric case attended a primary school, while the other two cases attended secondary schools. One of the adult cases was a teacher, while the other adult cases conducted educational sessions in schools that were up to 2 hours in duration. All cases except one had symptoms of either cough or fever in line with the European Centre for Disease Prevention and Control (ECDC) case definition for COVID-19 testing at the time [7]. One paediatric case was asymptomatic and was tested as part of the investigation of a household cluster. Their contacts are summarised in the Table. A total of 1,155 contacts of these six cases were identified. They were exposed at school in the classroom, during sports lessons, music lessons and during choir practice for a religious ceremony, which involved a number of schools mixing in a church environment.

**Table:** Cases of coronavirus disease with a history of school attendance and contacts, Ireland, 1 March–13 March 2020 (n = 1,160 individuals)

Case	Age group in years	Symptoms	Number of contacts				Number of secondary cases			
			Child		Adult		Child		Adult	
			School	Other^a^	School	Other^a^	School	Other^a^	School	Other^a^
1	10–15	Fever	475	29	30	3	0	0	0	0
2	10–15	None	125	30	25	8	0	0	0	0
3	10–15	Fever	222	14	28	0	0	0	0	0
4	Adult > 18	Coryza/cough	52	2	4	38	0	0	0	2
5	Adult > 18	Cough	39	2	2	3	0	0	0	0
6	Adult > 18	Cough	11	0	12	1	0	0	0	0

^a^ Other transmission settings include households of friends and family and recreational activities.

Among 1,001 child contacts of these six cases there were no confirmed cases of COVID-19. In the school setting, among 924 child contacts and 101 adult contacts identified, there were no confirmed cases of COVID-19.

### Contact tracing and follow-up

In line with Irish guidelines, contacts were defined as close contacts or casual contacts [8]. Close contacts were advised to restrict movements and underwent active surveillance with daily contact from Public Health monitoring for symptoms until 14 days from last exposure to a case. Casual contacts were advised to monitor for symptoms and given general information on physical distancing, hand hygiene and cough etiquette. Contacts who developed any symptoms consistent with COVID-19 were referred for testing. It was not possible to ascertain exact numbers of symptomatic contacts who were tested from records, however extensive testing was conducted. All symptomatic contacts (close or casual) were tested, even if only reporting mild symptoms of a respiratory tract infection. Although active follow-up of close contacts was conducted for 14 days from last exposure to a case, testing was not limited to this time period. Among all of the cases and contacts, transmission was observed in only one instance, which was outside the school environment, between two of the adult cases and a further adult.

### Ethical statement

This analysis was conducted as part of public health usual practice, and was not conducted for research. Ethics approval was therefore not needed.

## Discussion

In summary, examination of all Irish paediatric cases of COVID-19 attending school during the pre-symptomatic and symptomatic periods of infection (n = 3) identified no cases of onward transmission to other children or adults within the school and a variety of other settings. These included music lessons (woodwind instruments) and choir practice, both of which are high-risk activities for transmission. Furthermore, no onward transmission from the three identified adult cases to children was identified.

The only documented transmission that occurred from this cohort was between adults in a working environment outside school. Among 1,025 child and adult contacts of these six cases in the school setting there were no confirmed cases of COVID-19 during the follow-up period. Follow-up period was at least one incubation period (14 days) from last contact with a case. 

### Limitations

This study is limited by small numbers of cases. Not all age ranges are represented since all children are older than 10 years. During this time period there were no reported cases of outbreaks in childcare facilities, however younger children who did not attend school or childcare were not specifically included in this investigation.

Only symptomatic contacts were tested, and so asymptomatic secondary cases were not captured.

Prior to the nationwide closure of schools on 12 March, when a case was identified within a school, either all children and staff within the school or all children and staff involved with an individual case were excluded. This limited the potential for further transmission within the school setting once a case was identified. All contacts listed in the Table had been exposed to the cases before the schools closed however.

## Conclusion

While this study, based on small numbers, provides limited evidence in relation to COVID-19 transmission in the school setting, it includes all known cases with school attendance in the Republic of Ireland. The results moreover echo the experience of other countries, where children are not emerging as considerable drivers of transmission of COVID-19. Recent population screening studies from Iceland [9] and Italy [10] identified very few cases of COVID-19 disease in children with PCR testing. A report on school-related transmission in New South Wales, Australia, examining the spread of SARS-CoV-2 from 18 confirmed cases (nine students and nine staff) from 15 schools identified only two potential cases of secondary school-based transmission, despite the identification of 863 close contacts [11].

These findings suggest that schools are not a high risk setting for transmission of COVID-19 between pupils or between staff and pupils. Given the burden of closure outlined by Bayhem [4] and Van Lanker [5], reopening of schools should be considered as an early rather than a late measure in the lifting of restriction. Our report includes both the primary and secondary school setting, with no transmission in either setting. The limited evidence of transmission in school settings supports the re-opening of schools as part of the easing of current restrictions. There are no zero risk approaches, but the school environment appears to be low risk. 

On 10 March 2020, the United Nations Children’s fund (UNICEF), the International Federation of the Red Cross and the WHO issued a guidance document on re-opening schools [12]. The guidance considers the balance of risks to children’s health, well-being, learning and development posed by disease transmission vs not attending school. The document also states that marginalised children are likely to suffer more from school closures. In line with this and ECDC recommendations [13,14], countries can begin to lift restrictions once transmission within the community is controlled, there is surge capacity within the healthcare system and adequate resources are in place for active case finding, testing and contact tracing. Careful attention will still need to be paid to hygiene and respiratory etiquette, both in the classroom and in areas where staff congregate. Monitoring for and exclusion of staff or students with symptoms of respiratory illness and contact tracing would continue as normal. Public Health control measures will be put in place if individual cases within the school are identified, as is usual practice. If this is adhered to there is no reason to believe that the schools cannot be safely reopened.
